# Structural and Functional Alterations in The Zona Fasciculata of The Rat Adrenal Cortex in Obstructive Jaundice

**DOI:** 10.1155/1989/34079

**Published:** 1989

**Authors:** P. T. Roughneen, K. M. Tallon, Diane Haley Russell, B. J. Rowlands

**Affiliations:** The University of Texas Medical School Houston Texas USA

## Abstract

This study investigated the effect of experimentally-induced cholestasis in the rat on the structure and function of the zona fasciculata, the glucocorticoid secretory region of the adrenal cortex. Wistar-Furth rats (200-250g) were assigned to three groups: bile duct ligated (BDL), Sham operated (Sham) and unmodified normal control (NC). On day 14, serum bilirubin and liver histology were performed to confirm cholestasis in BDL animals together with basal 24 hour 17-hydroxycorti-costeroid excretion, adrenal histology and zona fasciculata ultrastructure in all experimental groups. Following this laparotomy, structural and functional studies were repeated on day 15 to evaluate the response of the gland to surgically induced stress. Basal 24 hr. 17-hydroxycorticoid steroid excretion was elevated in BDL animals (26.9 ±3.2 μ g/24 hr) with respect to Sham (10.4 ± 2.3) and NC groups (13.5 ± 3.2) (*p* <0.05). Adrenal histology and ultrastructural studies demonstrated excessive accumulation of vesicles laden with glucocorticoid biogenic precursors. Following surgical stress 24 hr 17 OH corticosteroid excretion increased in all groups: BDL (31.0 ± 3.0 μg/24 hr) *vs* Sham (15.6 ± 1.8) and NC (14.5 ± 2.4) Moderate alterations in zona fasciculata architecture were seen following surgery in all groups. Cholestasis induces overactivity of the zona fasciculata of the adrenal cortex, and may modify the normal metabolic responses to surgical and other stresses.


*HPB Surgery* 1989, Vol. 4, pp. 271–281
Reprints available directly from the publisher
Photocopying permitted by license only

© 1989 Harwood Academic Publishers GmbH
Printed in Great Britain

### STRUCTURAL AND FUNCTIONAL ALTERATIONS IN THE ZONA FASCICULATA OF THE RAT ADRENAL CORTEX IN OBSTRUCTIVE JAUNDICE

P.T. ROUGHNEEN, K.M. TALLON, DIANE HALEY RUSSELL and B.J. ROWLANDS

*The University of Texas Medical School Houston, Texas, USA*

*(Received 18 July 1988)*

This study investigated the effect of experimentally-induced cholestasis in the rat on the structure and function of the zona fasciculata, the glucocorticoid secretory region of the adrenal cortex. Wistar-Furth rats (200–250g) were assigned to three groups: bile duct ligated (BDL), Sham operated (Sham) and unmodified normal control (NC). On day 14, serum bilirubin and liver histology were performed to confirm cholestasis in BDL animals together with basal 24 hour 17-hydroxycorti-costeroid excretion, adrenal histology and zona fasciculata ultrastructure in all experimental groups. Following this laparotomy, structural and functional studies were repeated on day 15 to evaluate the response of the gland to surgically induced stress. Basal 24 hr. 17-hydroxycorticoid steroid excretion was elevated in BDL animals (26.9 ± 3.2 $\mu$ g/24 hr) with respect to Sham (10.4 ± 2.3) and NC groups (13.5 ± 3.2) ($p$ <0.05). Adrenal histology and ultrastructural studies demonstrated excessive accumulation of vesicles laden with glucocorticoid biogenic precursors. Following surgical stress 24 hr 17 OH corticosteroid excretion increased in all groups: BDL (31.0 ± 3.0 $\mu$g/24 hr) *vs* Sham (15.6 ± 1.8) and NC (14.5 ± 2.4) Moderate alterations in zona fasciculata architecture were seen following surgery in all groups. Cholestasis induces overactivity of the zona fasciculata of the adrenal cortex, and may modify the normal metabolic responses to surgical and other stresses.

KEY WORDS: Zona fasciculata, cholestasis, adrenal cortex, obstructive jaundice.

### INTRODUCTION

Patients with obstructive jaundice have a high incidence of postoperative morbidity and mortality. Several clinical studies have indicated important risk factors associated with the development of postoperative complications[1,2,3,4]. Such factors include low serum albumin, elevated serum bilirubin, elevated serum creatinine, decreased hematocrit, the presence of malignant disease and age greater than 60 years. These factors have been shown to correlate significantly with postoperative complications such as sepsis, renal impairment and gastrointestinal hemorrhage and occur in up to 30% of patients with obstructive jaundice undergoing operation. Despite this high incidence of complications, little is known of adrenocortical function in cholestasis. Integrity of the adrenocortical axis is critical for host survival in response to severe stress. It is well known that administration of exogenous glucocorticoids to patients with adrenal dysfunction is essential for host survival following an operative or other stressful stimuli[5]. The principal corticosteroid in man is cortisol, a steroid molecule produced by the zona fasciculata under the influence of ACTH stimulation by the adenohypothesis. Following secretion into the blood stream and elaboration of its systemic effects, cortisol is metabolized by the liver to 17-ketogenic steroid and excreted as conjugates of glucuronic acid and excreted in the urine[6,7]. In an attempt to determine if alterations in adrenocortical function could be a contributory factor to the high incidence of morbidity and mortality seen in cholestatic patients, we evaluated the effect of experimental cholestasis on the structural and functional integrity of zona fasciculata of the rat adrenal gland.

### MATERIALS AND METHODS

### *Animals*

Female Wistar Furth rats (200–250 g) (Harlan Sprague Dawley, Indianapolis, Indiana) were maintained at room temperature and subjected to a 12-hour artificial daylight cycle. Throughout the study animals were fed standard rodent chow (Purina Formulab 5008) and allowed tap water ad libitum. At day 0 animals were divided into three groups (Figure 1): bile duct ligated (BDL), Sham operated (Sham) or normal control (NC) and thereafter placed in individual metabolic cages (Wahmann, Timonium, MD). On day 14, urine was collected from study animals and 24-hour 17-hydroxycorticosteroid output determined. Rats were then either sacrificed for adrenal histology and ultrastructural studies, or underwent laparotomy with manipulation of the gastrointestinal tract under pentobarbital anesthesia (3.2 mg/100g body weight). This latter procedure was undertaken to simulate a moderate surgical stress in experimental animals. Following surgery, rats were returned to metabolic cages and 24-hour 17-hydroxycorticosteroid excretion determined on the first postoperative day. Animals were then sacrificed under pentobarbital anesthesia (10 mg/100g body weight) and adrenal glands removed for further histological and ultrastructural studies.

### *Bile Duct Ligation*

This was performed under pentobarbital anesthesia (3.2 mg/100g body weight) on the first day of the study: a central upper abdominal incision was made, the common bile duct identified, doubly ligated with two 3-0 silk ligatures, and divided[8]. High bile duct ligation was performed in the same position in all animals. In those animals

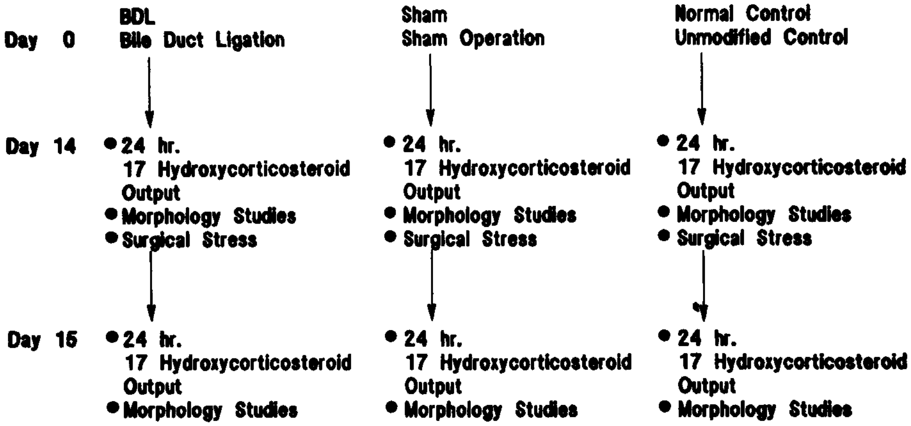

**Figure 1** Time schedule and experiment procedures in study animals.

undergoing sham operation, the common bile duct was identified and freed from the surrounding tissue only. The abdomen was then closed with 2/0 silk.

### *Surgically Induced Operative Stress*

Induction of an experimental operative stress involved a midline laparotomy with exteriorization of the bowel. Mesenteric traction was then applied for 5 minutes after which the bowel was returned to the abdominal cavity and the incision closed with 2/0 silk.

### *Serum Total Bilirubin and Liver Histology Following Bile Duct Ligation*

Serum total bilirubin was determined on day 14 following operation by the Jendrassik-Grof method (Animal Reference Laboratories, Houston, TX)[9]. Following sacrifice livers were removed from study animals and fixed in 10% Formalin for 48 hours. Semi-thin sections were then cut on a LKB Nova ultratome. The sections were then stained with haematoxylin and eosin and photographed with a Nikkon light microscope.

### *24-Hour Hydroxycorticosteroid Excretion*

Twenty-four hour urinary output was collected from animals in metabolic cages in 20 ml urinal vials. Urinary output was recorded and the 17-hydroxycorticosteroid content of mixed aliquots of samples determined by the Porter-Silber Method (Dept. of Laboratory Medicine, Hermann Hospital, Houston, TX). This assay is based on the formation of a yellow pigment (maximum absorption 410 nm) when certain corticosteroids react with phenylhydrazine in the presence of alcohol and sulphuric acid. The color reaction occurs primarily with corticosteroids that possess a dihydroxyacetone side chain. Corticosteroids with this configuration include cortisol, cortisone, 11-deoxycortisol and their tetrahydro-derivatives. In this assay, 2 ml of urine was added to test tubes and the pH adjusted to 6.8. Two hundred microliters of glucuronidase (1000 u/ml), 400 $\mu$l phosphate buffer (0.5 mol/1) and 20 $\mu$l of chloroform were then added to tubes and incubated at 37° C for 18 hrs. Samples were then mixed with 300 mg ammonium sulphate for 5 mins. and the washed sample incubated with Porter-Silber reagant for 30 mins. at 60° C. Absorbance was measured spectrophotometrically at 410 nm. using blanks to adjust to zero absorbance. Corticosteroid concentrations were then calculated from the following formula:

$$\text{Corticosteroid conc (mg/dl)} = \frac{\text{Absorbance (test)-Absorbance (blank)} \times 0.5}{\text{Absorbance (Standard)-Absorbance (Standard Blank)}}$$

### *Adrenal Morphology and Histology*

Following sacrifice both adrenal glands were immediately removed from the animal, dissected free of surrounding fascia, and partially bisected in a transverse plane into two equal halves. They were immersed in a fixative solution of 2 per cent formaldehyde and 1 per cent glutaraldehyde (2F-1G) in a 200 mOsm phosphate buffer, pH 7.4 for several minutes, blotted and weighed individually[10]. They were then returned to the fixative solution for three hours. A single midline transverse section 1 mm in thickness was removed from the left adrenal gland of each animal and returned to the fixative for 24 hours. Sections from all animals were examined and photographed on a Nikon dissecting microscope. They were rinsed in Millonig's phosphate buffer (pH 7.4), postfixed in 1 per cent phosphate buffered osmium tetroxide for two hours, stained en bloc in uranyl acetate for 30 minutes, dehydrated in a graded series of alcohols, treated with propylene oxide and embedded in epoxy resin. Semi-thin sections were cut on an LKB Nova ultratome yielding entire transverse sections inclusive of cortex and medulla. The sections were stained with toluidine blue and viewed and photographed with a Nikon light microscope. Specimens from stressed and non-stressed groups of rats were examined separately by one observer (D.H.R.) having no further knowledge of their prior treatment. The glands were placed into one of three histologically distinct subgroups based on subjective evaluation of lipid content.

### *Transmission Electron Microscopy Studies*

The same specimens prepared and embedded in epoxy were used for transmission electron microscopy studies. Ultra-thin sections of the tissue were cut, mounted on 300 mesh copper grids, and stained with lead citrate. These sections were examined and photographed in a JEOL JEM-CX 100 transmission electron microscope.

### *Statistical Analysis*

Results of biochemical studies were expressed as mean ± SEM and subjected to the Students unpaired "t" test. A p value less than 0.05 was considered statistically significant.

### RESULTS

### *Serum Total Bilirubin and Liver Histology*

Serum total bilirubin was significantly elevated in BDL animals with respect to Sham and NC groups at day 14:14.2 ± 0.5 *vs* 0.1 ± 0 and 0.1 ± 0 mg/dl, respectively. Gross morphological features of hepatomegaly, splenomegaly and portal venous engorgement were seen in BDL animals at the time of sacrifice. Liver histology demonstrated features of biliary proliferation, periportal fibrosis, and areas of minimal necrosis in BDL animals. These biochemical and morphological features in BDL animals demonstrate biochemical and structural alterations consistent with cholestatic liver disease.

### *24-Hour 17-Hydroxycorticosteroid Excretory Studies*

Fourteen days following bile duct ligation, jaundiced rats excreted significantly greater amounts of 17-hydroxycorticosteroid than Sham operated and normal control animals ($p < 0.05$) (Table 1). Following an operative stress, animals in the basal cholestatic state demonstrated increased excretion of 17-hydroxycorticosteroid metabolites compared to pre-operative values, although this increase was not

**Table 1** 17-hydroxycorticosteroid output (mean ± SEM — μ g/24hr) in BDL, Sham, and NC rats pre- and post-laparotomy.

| | *BDL* | *Sham* | *NC* |
|---|---|---|---|
| Pre-laparotomy | 26.9 ± 3.2* (n = 13) | 10.4 ± 2.3 (n = 16) | 13.5 ± 3.2 (n = 18) |
| Post-laparotomy | 31.0 ± 3.0† (n = 10) | 15.6 ± 1.8 (n = 11) | 14.5 ± 2.4 (n = 15) |

* BDL vs Sham and NC $p<0.05$

† BDL vs Sham and NC $p<0.05$

Prelaparotomy vs Post laparotomy — all groups — NS — not significant statistically significant ($p < 0.1$). Furthermore, 17-hydroxycorticosteroid output remained elevated in BDL animals with respect to surgically stressed Sham and NC groups ($p < 0.05$). These results suggest that the cholestatic state induces hyperactivity of the adrenal cortex as reflected in the increased excretion of glucocorticoid metabolites. Furthermore, glucocorticoid metabolite excretion remains elevated, with no evidence of failure of vital glucocorticoid responses in the surgically stressed state.

### *Morphological, Histological and Ultrastructural Studies of the Zona Fasciculata*

There were no differences in the ratio of adrenal: carcass weight among experimental groups (Table 2). Sections of adrenal glands from BDL, Sham and NC animals were easily distinguished macro- and microscopically from one another, although there were no consistent differences among individual glands with regard to size or cortical width. In both stressed and non-stressed groups, the cortical region of each BDL animal was noticeably lighter in color than that of sham operated rats, which in turn was slightly lighter than the adrenal cortex of normal rats (Figure 2). The reason for this color differentiation was readily apparent when viewing the section with light microscopy. The adrenal glands fell into three distinct groups based on lipid accumulation within the cortex (Figures 3-5). In normal rats, lipid droplets were distributed throughout all zones of the adrenal cortex, although they were slightly more numerous in the zona fasciculata (ZF) than the zona glomerulosa (ZG), and least apparent in the zona reticularis (ZR). Considerable heterogeneity existed among the cells in any one zone with regard to the quantity and size of the lipid inclusions as can be seen in the ZF of a normal control animal (Figure 3) In Sham operated

**Table 2** Adrenal gland to body weight ratio (× 1000) (mean ± SEM) in BDL, Sham, and NC rats when non-stressed and stressed.

| | *BDL* | *Sham* | *NC* |
|---|---|---|---|
| Non-stressed | 158 ± 10 | 176 ± 6 | 161 ± 5 |
| Stressed | 209 ± 23 | 186 ± 6 | 179 ± 9 |

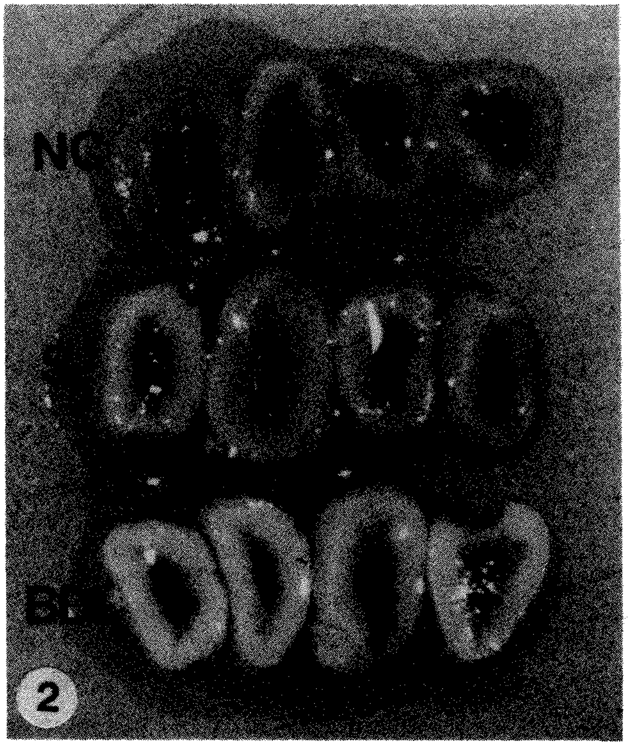

**Figure 2** Gross features of adrenal glands from non-stressed (day 14) normal control (NC), sham (S), and bile-duct ligated (BDL) animals. Although there are no consistent differences among the glands in size, cortexes of BDL animals are strikingly lighter than those of the other groups. Cortexes of sham animals are slightly lighter than those of normal controls. X 7.

animals, there was a marked increase in the size and number of lipid droplets in all three cortical zones, being most pronounced in the ZF (Figure 4). In glands from BDL animals, lipid accumulation was very pronounced in all layers. Cells of the ZF were lipid laden to the extent that they appeared foamy at low magnifications (Figure 5). A representative example of the ultrastructural features of lipid accumulation in the fasciculata cells of cholestatic animals is demonstrated in Figure 6. Following a surgically induced stress ZF lipid accumulation appeared to be more pronounced in each subgroup of the stressed rats relative to the equivalent subgroup of unstressed rats. No changes were detected in the adrenal medulla of any of the animals in either the non-stressed or stress states.

### DISCUSSION

The predisposition of the cholestatic host to increased postoperative morbidity and mortality is well documented. The major postoperative complications leading to increased mortality and morbidity seen in these patients are renal failure, sepsis and gastrointestinal hemorrhage. Although the propensity of the cholestatic host to postoperative complications is well established in the literature, little is known of the

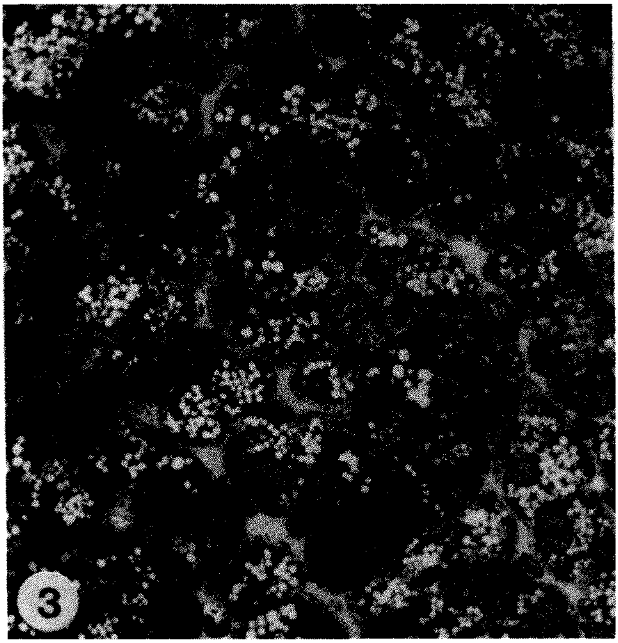

**Figure 3** Light microscopy of the zona fasciculata of a normal control (NC) animal. Lipid is variably distributed throughout the cytoplasm of many cells and absent in others. X 100.

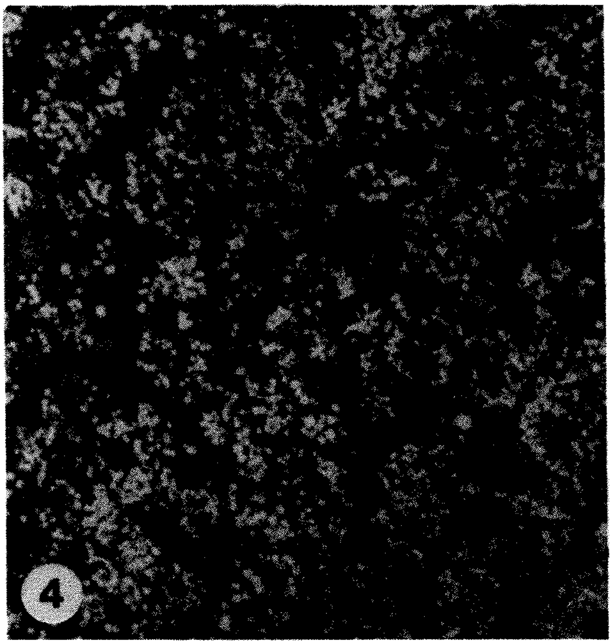

**Figure 4** Light micrograph of the zona fasciculata from a sham (S) rat 14 days after simple manipulation of the common bile duct. Lipid is present in virtually all cells. X 1000.

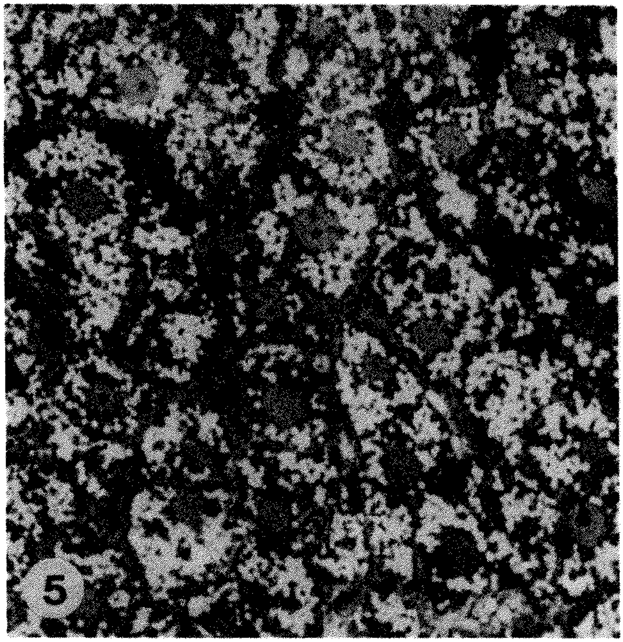

**Figure 5** Light micrograph of the zona fasciculata from a rat 14 days after ligation of the common bile duct (BDL). The cytoplasm of most cells is filled with lipid droplets, giving them a foamy appearance. X 1000.

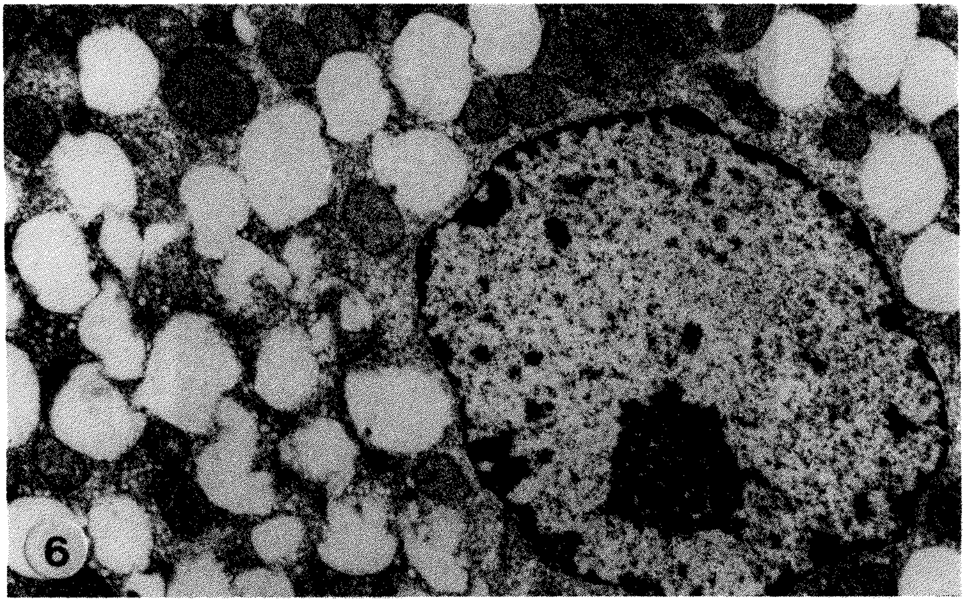

**Figure 6** Transmission electron micrograph of a fasciculata cell from a BDL rat demonstrating abundant clear lipid droplets, mitochondria and smooth endoplasmic reticulum interspersed throughout the cytoplams. X 8100.

effect of cholestasis on adrenocortical function, critical for host survival following surgical and other pathological stresses. The fundamental role of optimal adrenal function in the metabolic response to surgical stress led us to investigate the structural and functional integrity of the zona fasciculata in experimental cholestasis in both basal and stressed conditions. The principal glucocorticoid in man is cortisol, a molecule that has important effects on carbohydrate metabolism including promotion of gluconeogenesis, deposition of liver glycogen, and elevation of blood glucose due to decreased carbohydrate utilization. Glucocorticoids also inhibit amino acid uptake and protein synthesis in the peripheral tissues and affect lipid metabolism by stimulating fat synthesis. In addition to their effect on intermediary metabolism, glucocorticoids exert essential permissive effect in maintaining vascular reactivity to catecholemines, preventing muscle fatigue and aiding renal excretion of a water load. Glucocorticoids are also well known for their non-selective immunosuppressive effect resultant from a diminution in the number of circulating immunocompetent cells and the gradual destruction of lymphoid tissue. In view of the vital role of adrenocortical function in the regulation of metabolic homeostasis under both resting and stressful conditions, we were interested to evaluate the effect of cholestasis on the integrity of the rat zona glomerulosa.

The data demonstrates a striking effect of coexisting cholestasis on both the functional and structural integrity of the zona fasciculata. Cholestatic animals excreted large amounts of 17-hydroxycorticosteroid over a 24-hour period suggestive of increased functional activity of the zona fasciculata. This functional alteration was reflected in the morphological appearance of the glands in the accumulation of excessive lipid in the adrenal cortex. Light microscopy demonstrated distention of fasciculata cells with clear round vesicles of variable size. Transmission electron microscopy confirmed the presence of numerous lipid droplets, mitachondria and smooth endoplasmic reticulum. These droplets or vesicles are known to contain cholesterol, the biogenic steroid precursor of glucocorticoid synthesis. The morphological alterations in the zona fasciculata provide structural evidence which, in accordance with the enhanced 24-hour hydroxycorticoid steroids excretion studies demonstrate excessive glucocorticoid synthesis by this region in cholestasis. It was interesting to note that sham operated animals exhibited moderate changes in adrenal morphology compared to normal control animals, although this was far less pronounced than that seen in cholestatic animals and was not reflected in 17-hydroxycorticosteroid excretion studies. This effect appears to be due to stimulation of the adrenal gland resulting from surgical stress alone. Further studies designed to examine adrenocortical function in the stressed cholestatic host demonstrated that cholestatic animals were able to increase their glucocorticoid output, although the differences were not significant from preoperative values. Sham and normal control animals also demonstrated increases in their 17-hydroxycorticosteroid output in response to surgical stress. These results demonstrate that although cholestatic animals did not significantly increase their 17-hydroxycorticosteroid output in response to moderate stress, the hyperfunctioning adrenal gland can at least maintain basal secretory levels and does not fail in response to such moderate stress.

The data may have pertinent clinical implications. We have demonstrated that experimental cholestasis results in increased metabolic activity of the zona fasciculata as reflected by increased glucocorticoid secretion and altered adrenal morphology. This poses the question as to whether such alterations in adrenocortical function similarly occur in patients with cholestatic disease. If so, the increased demand for glucocorticoid synthesis induced by major surgical or non-surgical stresses may not be fully met by the already exhausted and hyperfunctioning gland resulting in the demise of the host. Increased postoperative mortality among cholestatic patients is well documented clinically and is very common in experimental studies on the cholestatic host. In addition to its important effects on intermediary metabolism glucocorticoids are also known to dampen normal host immunoresponsiveness. Studies by this group and others have demonstrated severe impairment of cellular immune responses in the cholestatic host[12,13,14]. It has been proposed that such alterations in host immunity may account for the well known propensity of the cholestatic host to develop infective complications. Elevated glucocorticoid synthesis by the zona fasciculata in cholestasis may be responsible for this important derangement of immune responses. High basal levels of serum glucocorticoids may inhibit normal host immunosuppressiveness to infective pathogens *in vivo*, thereby explaining the high incidence of sepsis in cholestasis. Studies by this group have so far failed to demonstrate transferrable serum immunosuppressive factors in cholestatic sera *in vitro* however. It is possible that alterations in adrenocortical function similarly occur in other chronic liver diseases such as cirrhosis[15]. For instance, it is well known that cirrhotic liver disease can result in secondary hyperaldosteriodism in the host. In addition to effects on the zona glomerulosa it is possible that structural and functional alterations in the zona fasciculata, similar to those seen in experimental cholestasis, may also occur in the cirrhotic subject. It is of interest that cirrhotic patients share many of the pathological sequelae seen in the cholestatic host following surgical intervention.

Clearly, further studies are required to determine if the reported alterations in the functional integrity of glucocorticoid mediated responses are beneficial or harmful to the cholestatic host. When this important information is available, it will be possible to employ the most suitable form of therapeutic intervention in order to improve the prognosis of this patient cohort. If the excessive glucocorticoid synthesis by the zona fasciculata is beneficial to the cholestatic host then it is possible that additional administration of exogenous glucocorticoids may improve the host's metabolic response to surgical and other stresses. If, however, excessive circulating levels of glucocorticoid are detrimental to the diseased host then therapeutic restoration of hepatic function by biliary drainage may restore adrenocortical function to levels seen in normal subjects and thereby reverse this potentially detrimental alteration in host metabolism.

### *References*

1. Pitt, H.A., Cameron, J.L., Postier, R.G. and Gadacz, T.R. (1981). Factors affecting mortality in biliary tract surgery. *Am. J. Surg.*, **141,** 66–72
2. Blamey, S.L., Fearon, K.C.H., Gilmour W.H., Osborne, D.H. Carter, D.C. (1983).Prediction of risk in biliary surgery. *Br. J. Surg.*, **70,** 535–538
3. Armstrong C.P., Dixon J.M., Taylor T.V., Davies G.C. Surgical experience of deeply jaundiced patients with bile duct obstruction. *Br. J. Surg.*, (1984), **71,** 234–238
4. Hunt D.R. 1980; The identification of risk factors and their application to the management of obstructive jaundice. *Aust N.Z. J. Surg.*, **50,** 476–480
5. Kaplan, E.L., Lee, R.C. (1980); Acute postoperative hormonal insufficiency syndromes. In: Condon R.E., Decosse, J.J. *Surgical Care — A physiological approach to clinical management,* Philadelphia, Lea and Febiger, 370–384
6. Williams, G.H., Dluhy, R.G. (1983); Diseases of the adrenal cortex. In: Petersdorf R.G., Adams,

R.D., Brawnwald, E., Isselbacher K.J., Martin, J.B., Wilson, D., Eds. *Harrisons principles of internal medicine.* New York: *McGraw Hill,* 634–657
7. Chattoraj, S.C. (1976); Endocrine Function. In: Tietz N. Fundamentals of clinical chemistry. Philadelphia: *Saunders* 727–730
8. Lee E. (1972); The effect of obstructive jaundice on the migration of reticuloendothelial cells and fibroblasts into early experimental granulomata. *Br. J. Surg.,* **59,** 875–877
9. Walters M.A., Gerarde, H.W. (1970); An ultramicromethod for the determination of conjugated and total bilirubin serum or plasma. *Microchem, J.,* **15,** 231–243
10. McDowell, E., Trump, B.F. (1976); Histological fixatives suitable for light and electron microscopy. *Arch Pathol Lab Med.,* **100,** 405–414
11. Robbins, S.L., Cottran, R.S. (1979); In: Robbins S.L., Cottran, R.S., Eds. *Pathologic basis of disease.* Philadelphia: *Saunders,* 1387–101
12. Fargion, S.R., Podda, M., Cappellini, M.D., *et al.* (1976); Immunita cellulare nelle celestasi intra ed extra-epatriche. *Minerva Gastroenterologica* **22,** 261–265
13. Pinto, M., Kaplun, A. (1980); Immune status in mice with experimental biliary obstruction. *Clin. Immunol Immunopathol.,* **16,** 396–405
14. Katz, S., Grosfield, J.L., Gross, K., *et al.* (1984); Impaired bacterial clearance and trapping in obstructive jaundice. *Ann. Surg.,* **199,** 14–20
15. Garrison, R.N., Cryer, H.M., Howard, D.A., Polk, H.C. (1984); Clarification of risk factors for abdominal operations in patients with hepatic cirrhosis. *Ann. Surg.* **199,** 648–654

